# Photoprotection Is Achieved by Photorespiration and Modification of the Leaf Incident Light, and Their Extent Is Modulated by the Stomatal Sensitivity to Water Deficit in Grapevines

**DOI:** 10.3390/plants11081050

**Published:** 2022-04-12

**Authors:** Luis Villalobos-González, Nicolás Alarcón, Roberto Bastías, Cristobal Pérez, René Sanz, Álvaro Peña-Neira, Claudio Pastenes

**Affiliations:** Facultad de Ciencias Agronómicas, Universidad de Chile, Santiago 8820808, Chile; luisvillalobosg1@gmail.com (L.V.-G.); nicolasalarconvera.agro@gmail.com (N.A.); roberto.bastias.silva@gmail.com (R.B.); cristobal.perez@ug.uchile.cl (C.P.); rene.sanz@ug.uchile.cl (R.S.); apena@uchile.cl (Á.P.-N.)

**Keywords:** grapevines, light interception, water stress, photosynthesis, photorespiration, photoinhibition

## Abstract

Absorbed energy in excess of that used by photosynthesis induces photoinhibition, which is common in water deficit conditions, resulting in reductions in stomatal conductance. In grapevines, controlled water deficit is a common field practice, but little is known about the impact of a given water shortage on the energy transduction processes at the leaf level in relation to contrasting stomatal sensitivities to drought. Here, we assessed the effect of a nearly similar water deficit condition on four grapevine varieties: Cabernet Sauvignon (CS) and Sauvignon Blanc (SB), which are stomatal sensitive, and Chardonnay (CH) and Carménère (CM), which are less stomatal sensitive, grown in 20 L pots outdoors. Plants were maintained to nearly 94% of field capacity (WW) and 83% field capacity (WD). We have assessed plant water status, photosynthesis (A_N_), photorespiration, A_N_ vs. PAR, AC_i_ curves, photochemical (qP) and non-photochemical (qN) fluorescence quenching vs. PAR, the photoprotective effectiveness of NPQ (qPd) and light interception by leaves. Photorespiration is important under WD, but to a different extent between varieties. This is related to stomatal sensitivity, maintaining a safe proportion of PSII reaction centres in an open state. Additionally, the capacity for carboxylation is affected by WD, but to a greater extent in more sensitive varieties. As for qN, in WD it saturates at 750 μmol PAR m^−2^s^−1^, irrespective of the variety, which coincides with PAR, from which qN photoprotective effectiveness declines, and qP is reduced to risky thresholds. Additionally, that same PAR intensity is intercepted by WD leaves from highly stomatal-sensitive varieties, likely due to a modification of the leaf angle in those plants. Pigments associated with qN, as well as chlorophylls, do not seem to be a relevant physiological target for acclimation.

## 1. Introduction

Grapevines for oenological purposes are grown under controlled water deficit as a mean for increasing the grape berry quality [1,2], because of its effect on the berry size, microclimate of the fruiting zone and secondary metabolism [3,4,5,6].

Water stress, however, leads to deleterious effects. In fact, water is considered the essential environmental factor affecting plant productivity [7]. In general, plants react to water depletion by reducing their stomatal conductance (gs), which, in turn, leads to reductions in the mesophyll conductance to CO_2_ [8]. As water stress develops in a severe condition, further limitations to photosynthesis arise in plants, associated with damage to photosystems resulting from the light being absorbed in excess of the CO_2_ reduction capacity [9]. In addition, water-stress limitations of the CO_2_ with respect to that needed for CO_2_ reduction in photosynthesis have been reported, affecting Rubisco activity and/or regeneration of the Ribulose-biphosphate, as well as causing impairment in photophosphorylation [10,11,12]. Generally, the water stress effects on photosynthesis resulting from stomatal limitations have been distinguished from those coming from limitations involving the abovementioned biochemical impairments, as well as those arising from transient or permanent damage, and are called stomatal and non-stomatal limitations to photosynthesis, respectively [13]. In grapevines, these effects have been shown to correlate with gs, where declines down to 150 mmol H_2_O m^−2^s^−1^ are considered mild and reversible and, under an extreme situation, gs values below 50 mmol H_2_O m^−2^s^−1^ would result in non-reversible limitations [14].

On the other hand, it has been recognised that the energy absorbed in excess of that used in photochemistry might be harmful, leading to photoinhibition, which is defined as the inhibition of photosynthesis induced by strong intensity light in photosynthetic organisms [15]. Therefore, environmental constraints reducing the capacity for carbon reduction with no effect on light absorption are likely to induce photoinhibition [15]. Plants have evolved mechanisms for photoprotection, of which the non-radiative de-excitation through safe dissipation as heat, at the antenna chlorophylls, has been pointed out as the most significant [15,16]. Other potential mechanisms protecting photosynthesis, such as photorespiration, have been much less explored. For instance, by means of barley mutants with reduced activities of photorespiratory enzymes, it was suggested that photorespiration is enhanced by drought stress [17]. Indeed, and even though photorespiration is usually accounted as wasteful, because of the use of light in releasing previously fixed carbon, one photorespiratory cycle producing 0.5 phosphoglycerate molecules consumes 3.5 ATP and two NADH equivalents [18]. In other words, photorespiration may act as a safety valve when the energy pressure on the photosynthetic apparatus is increased, as in water stress-induced reductions in stomatal conductance.

Regarding stomatal responses to water availability, it is well accepted that differences exist between grapevine varieties. Controversies have arisen, however, regarding the midday stem water potential (Ψ_stem_) regulation in grapevines because of the difficulty in distinguishing the isohydric to anisohydric behaviour between varieties [19], the inconsistent behaviour in responses to drought reported for a given variety [20,21,22,23,24] and also because the iso or anisohydric behaviour has been argued to result from a plant–environment interaction, rather than an intrinsic property of the plant [22,25,26]. Yet, despite these discrepancies, a differential sensitivity to water deficit at the stomatal level, between varieties, is generally accepted [27].

Avoidance of the incident light is also important for preventing light-induced damage, particularly under water deficit. Leaf movements away from light have been mainly documented in leguminous plants, but this is a reversible mechanism triggered in the order of minutes [28,29], with the aid of specific morphological structures that are non-existent in grapevine leaves. However, more recently, the notion that grapevines change their leaf angle has been investigated and a relationship has been established with the stomatal conductance of the leaves [30].

As mentioned above, water stress—which is common in viticulture—leads to reductions in stomatal conductance, limiting carbon assimilation and increasing the risk of photodamage. Such effects, and the concomitant photoprotective mechanisms eventually deployed at the leaf level, depend on the stomatal sensitivity. That is, at any given shortage in soil water content, more stomatal sensitive varieties would have to respond to a higher extent in terms of photoprotection compared to less sensitive varieties. Thus, the aim of this study was to evaluate the relationship between the degree of the stomatal sensitivity with the extent of the responses triggered by mild water stress, at the level of photosynthetic gas exchange, photoprotection and light interception in the grapevine varieties Carménère (CM), Chardonnay (CH), Sauvignon Blanc (SB) and Cabernet Sauvignon (CS). These varieties have been chosen because they are among the most planted varieties in the world—except for CM, an emblematic variety in Chile—and also because of their known contrasting stomatal sensitivity to water stress [21,23]. To seek eventual patterns in the responses, the experiment was carried out in potted plants, frequently watered by weight, maintaining a well-irrigated treatment and a mild water-stress counterpart. We discuss the results regarding stomatal sensitivity with photorespiration, non-photochemical energy dissipation and light avoidance.

## 2. Results

### 2.1. Weather Conditions

**Figure 1:**
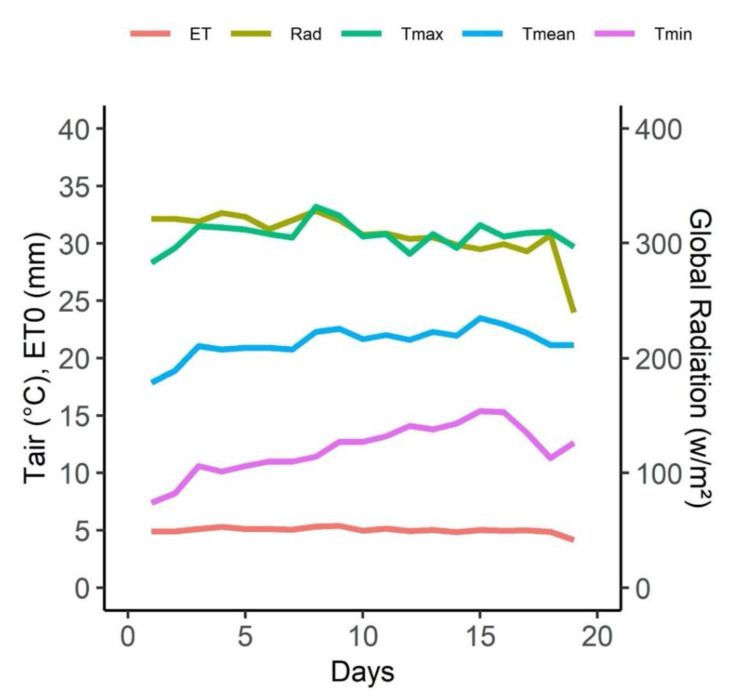
Climate variables during experiment. Daily values of minimum (Tmin, pink line), maximum (Tmax, dark green line) and mean (Tmean, blue line) temperature, daily reference evapotranspiration (ET, red line) and daily mean global radiation (Rad, light green line) through assay.

The experiment was carried out in midsummer with maximum temperatures higher than 30 °C, clear skies, and a daily reference evapotranspiration of nearly 5 mm, which is typical of Mediterranean climates. Minimum temperatures, on the other hand, varied between 7 and 14 °C (Figure 1).

### 2.2. Plant Water Status

**Figure 2:**
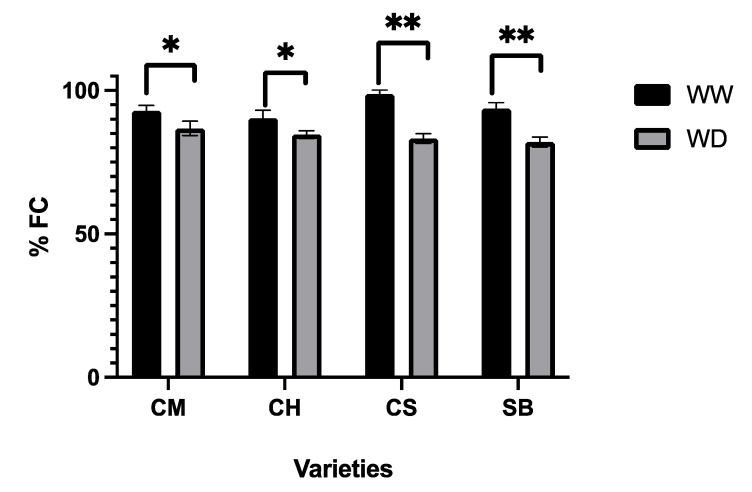
Average water content, as Field Capacity (FC) for WW and WD on Carmenere (CM), Chardonnay (CH), Cabernet sauvignon (CS) and Sauvignon blanc (SB). Single asterisk represents significant differences between treatments for each variety *p* ≤ 0.05. Double asterisk represents significant differences between treatments for each variety *p* ≤ 0.01.

**Figure 3:**
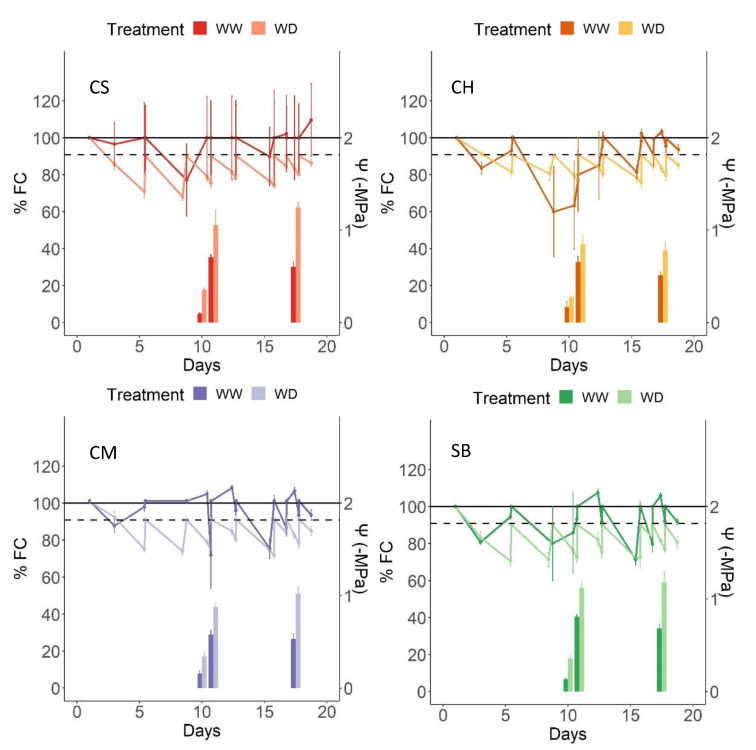
Water content in pots, determined by weight, for WW and WD plants, measured three times a week, along the experimental period for CM, CH, SB and CS. Also, at day 10, Ψ _pre-down_ and Ψ _stem_ and, at day 17, Ψ _stem_ are represented by paired bars. Light colour indicates WW and dark colour indicates WD. Upper horizontal line represents 100% of FC, and dotted line represents 90% of FC. Error bars represents SE.

As shown in Figure 2, the average soil water content of all the varieties, expressed as percentage of filed capacity (FC), corresponded to values from 90% FC to 98% FC in WW plants, and 84% FC to 87% FC in WD plants. The oscillation of average pot weight is shown in Figure 3, for WW and WD on each variety, showing higher daily weight, in general, for the WW pots. As expected from a low retention substrate, those minor differences resulted in contrasting and significantly different water status between treatments (Table 1). About 10 days from the beginning of the irrigation treatments, Ψ_pd_ values ranged from −0.09 MPa to −0.15 MPa in WW and from −0.27 MPa to −0.36 MPa, while Ψ_stem_ values ranged from −0.65 to −0.8 MPa and from −0.85 MPa to −1.1 MPa in WW and WD plants, respectively (Figure 2). These values were very similar at the end of the irrigation treatments; WW plants reached average Ψ_stem_ values of −0.6 MPa, −0.55 MPa, −0.5 MPa and −0.65 MPa for CS, CM, CH and SB, respectively. As for the WD plants, Ψ_stem_ values corresponded to −1.2 MPa, −1.0 Mpa, −0.8 MPa and −1.2 MPa for CS, CM, CH and SB, respectively (Figure 2). Regarding varieties, no significant differences were observed in Ψ_pd_ 10 days after the beginning of the irrigation treatments. CH and CM, on the other hand, presented more positive Ψ_md_ values after 10 days of irrigation treatments, as well as at the end of the experiment, with significant higher values for WW compared to WD. No interaction was observed between treatments (Table 1).

#### Photosynthesis, Photorespiration and Chlorophyll Fluorescence

**Figure 4:**
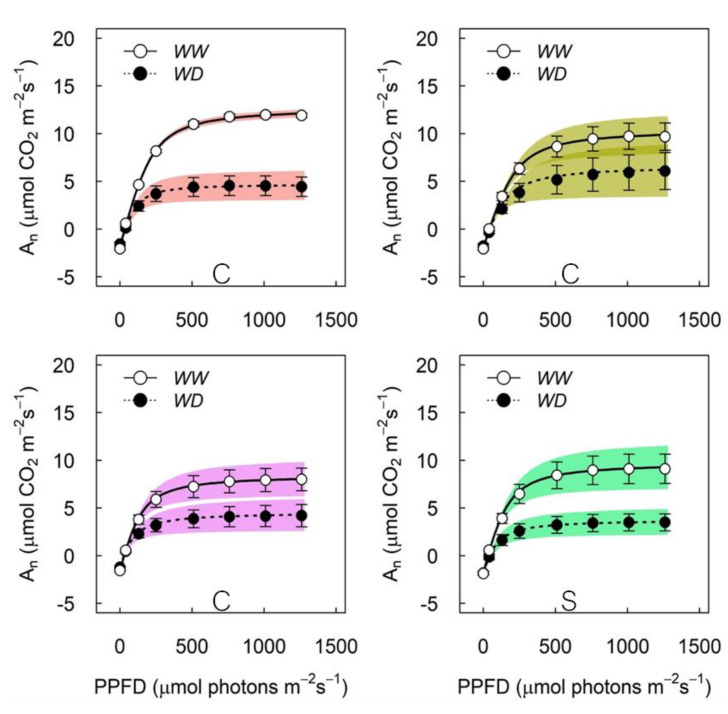
Net CO_2_ assimilation responses to light intensity for CM, CH, SB and CS. Dark Colour corresponds to WW and light colour to WD. Error bars represent SE and shaded area is the 95% confidence interval for the mean.

**Figure 5:**
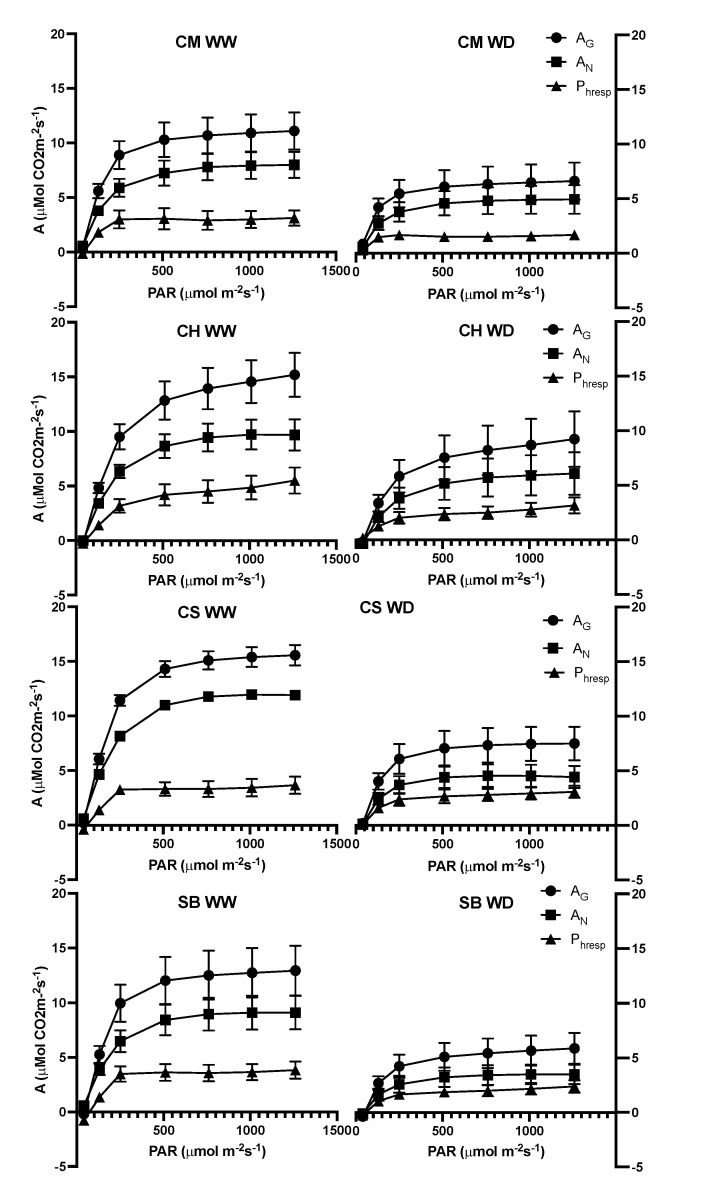
Gross net CO_2_ assimilation: AG; net CO_2_ assimilation: AN and photorespiration: Phresp responses to light intensities for Carmenere (CM), Chardonnay (CH), Cabernet sauvignon (CS) and Sauvignon blanc (SB) for WW (right figures) and WD (left figures). Error bars represent SE. Single asterisk represents significant differences between treatments for each variety *p* ≤ 0.05. Double asterisk represents significant differences between treatments for each variety *p* ≤ 0.01.

**Figure 6:**
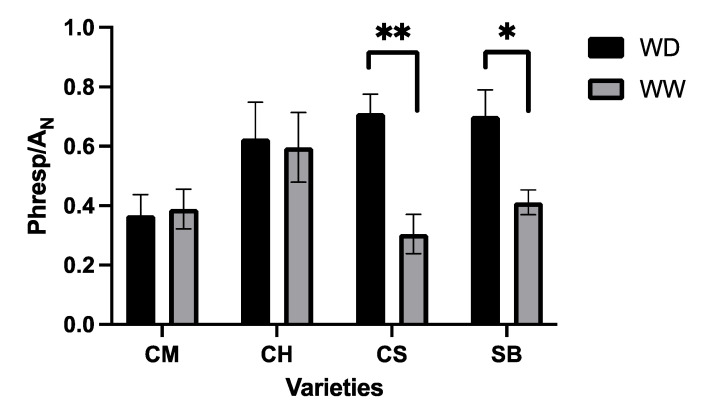
Relationship between photorespiration and net CO_2_ assimilation for CM, CH, CS and SB where black is WD and grey is WW. Error bars represent SE. Single asterisk represents significant differences between treatments for each variety *p* ≤ 0.05. Double asterisk represents significant differences between treatments for each variety *p* ≤ 0.01.

From the light-response curves, it was observed that WD plants reach lower values for maximal A_N_ compared to their WW counterparts, but to a different extent depending on the variety (Figure 4). The extent of reduction in the light-saturated An in WD plants, compared to WW, was 60% and 62% in CS and SB, respectively, much higher than the 40% reduction observed in CM and CH (Figure 4). At saturating light, differences in gs were observed between varieties, with higher values in CH and CS and lower in CM (Table 1). Additionally, at saturating light, the gs and A_N_ were significantly higher in WW compared to WD plants, with no interaction between treatments (Table 1). Figure 5 shows the light responses of A_N_, but also A_gross_ and the difference between both, interpreted as photorespiration. A_N_, for all the varieties and irrigation treatments, matched with the values observed from Figure 4. A_N_ and A_gross_ saturated at approximately 750 μmol photons m^−2^s^−1^ in all the varieties, irrespective of the irrigation treatment. In contrast, photorespiration also saturated at approximately 250 μmol photons m^−2^s^−1^ in WW and WD plants for all the varieties (Figure 5). Compared to A_N_, photorespiration was strongly increased by WD at saturating light, more than proportional to A_N_, in CS and SB, but not in CM and CH, where the photorespiration to A_N_ ratio was similar for both irrigation treatments (Figure 6).

**Figure 7:**
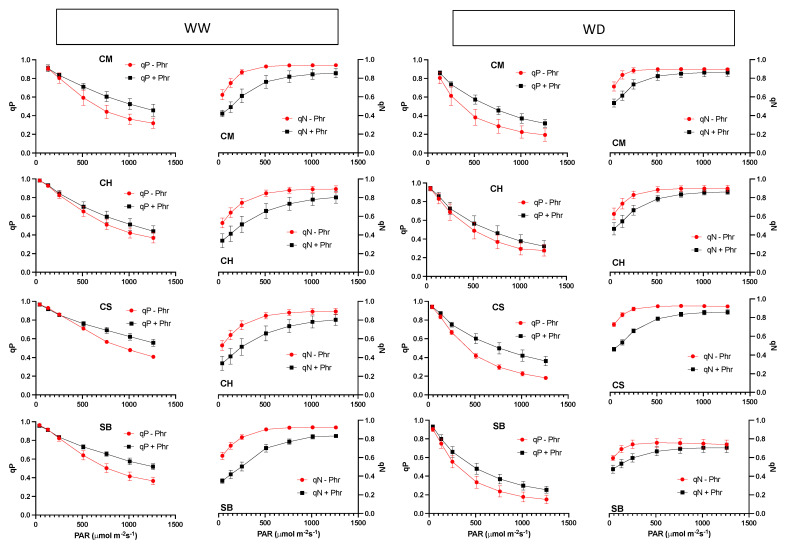
Photochemical (qP) and non-photochemical (qN) quenching responses of WW plants (two panels to the left) and WD plants (two panels to the right) to light intensity in photorespiratory conditions (+Phr: squares, black colour) and non-photorespiratory conditions (-Phr: circles, red colour) in Carmenere (CM), Chardonnay (CH), Cabernet sauvignon (CS) and Sauvignon blanc (SB). Error bars represent SE.

In general, qP is reduced as light increases, but at lower rates at intensities of 750 μmol photons m^−2^s^−1^ and higher, in all the varieties and irrigation treatments, and in photorespiratory as well as in non-photorespiratory conditions (Figure 7). However, qP reaches lower average values at high light intensities under non-photorespiratory conditions (Figure 7). As for qN, and opposite to qP, values saturate at light intensities of 750 μmol photons m^−2^s^−1^ in the WW plants under non-photorespiratory conditions, but at slightly higher intensities under photorespiratory air in all the varieties (Figure 7). In WD under photorespiratory conditions, qN saturates at light intensities of 750 μmol photons m^−2^s^−1^, approximately, while under non-photorespiratory conditions qN saturates at light intensities of nearly 500 μmol photons m^−2^s^−1^ in all the varieties (Figure 7).

**Figure 8:**
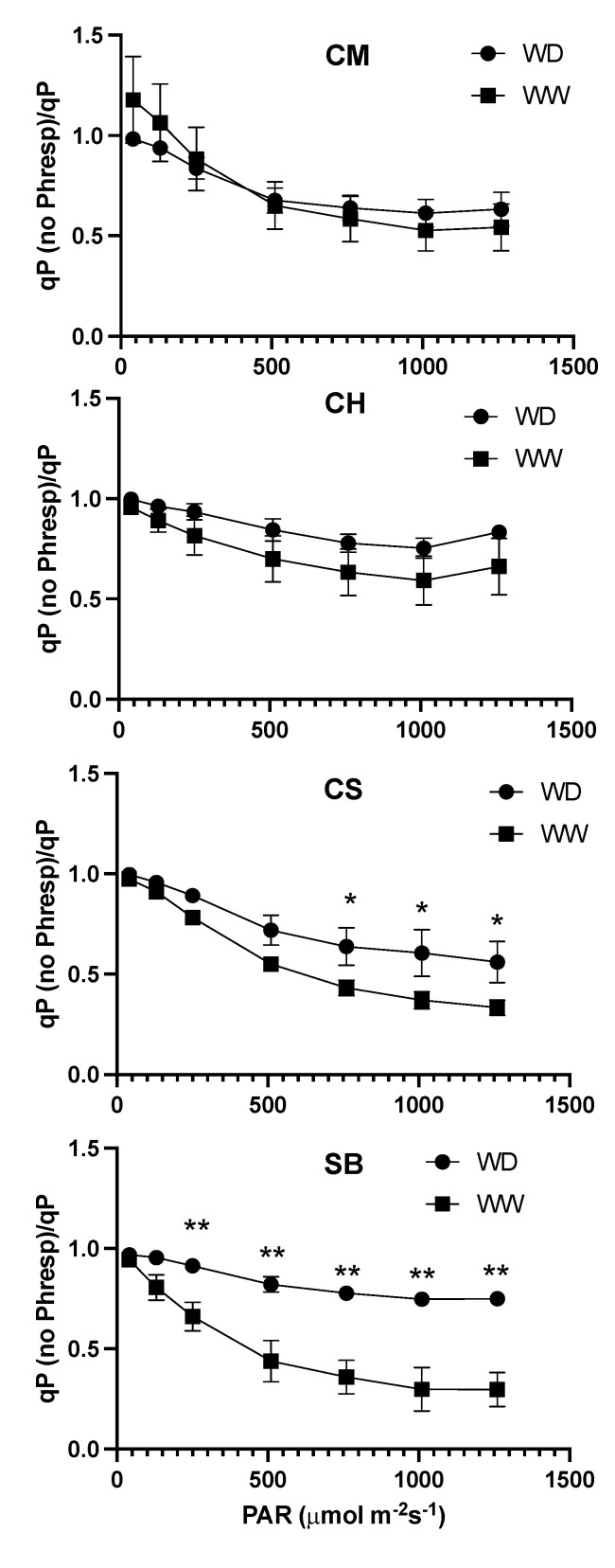
Relationship between qP measured under non-photorespiratory conditions and under photorespiratory conditions, at increasing light intensities in WW (squares) and WD (circles) for Carmenere (CM), Chardonnay (CH), Cabernet sauvignon (CS) and Sauvignon blanc (SB). Error bars represents SE. Single asterisk represents significant differences between treatments for each variety *p* ≤ 0.05. Double asterisk represents significant differences between treatments for each variety *p* ≤ 0.01.

The ratio between qP under non-photorespiratory conditions vs. photorespiratory conditions is shown in Figure 8. In the case of CM and CH, no significant differences were observed between irrigation treatments. In CS, differences in light intensities were significant: 750 μmol photons m^−2^s^−1^ and higher. In SB WW and WD, the ratio was significantly different at light intensities of 250 μmol photons m^−2^s^−1^ and higher, in both cases with higher values in WD plants (Figure 8). Differences between varieties were significant, as well as between irrigation treatments, but with no interaction between both factors (Table 2).

**Figure 9:**
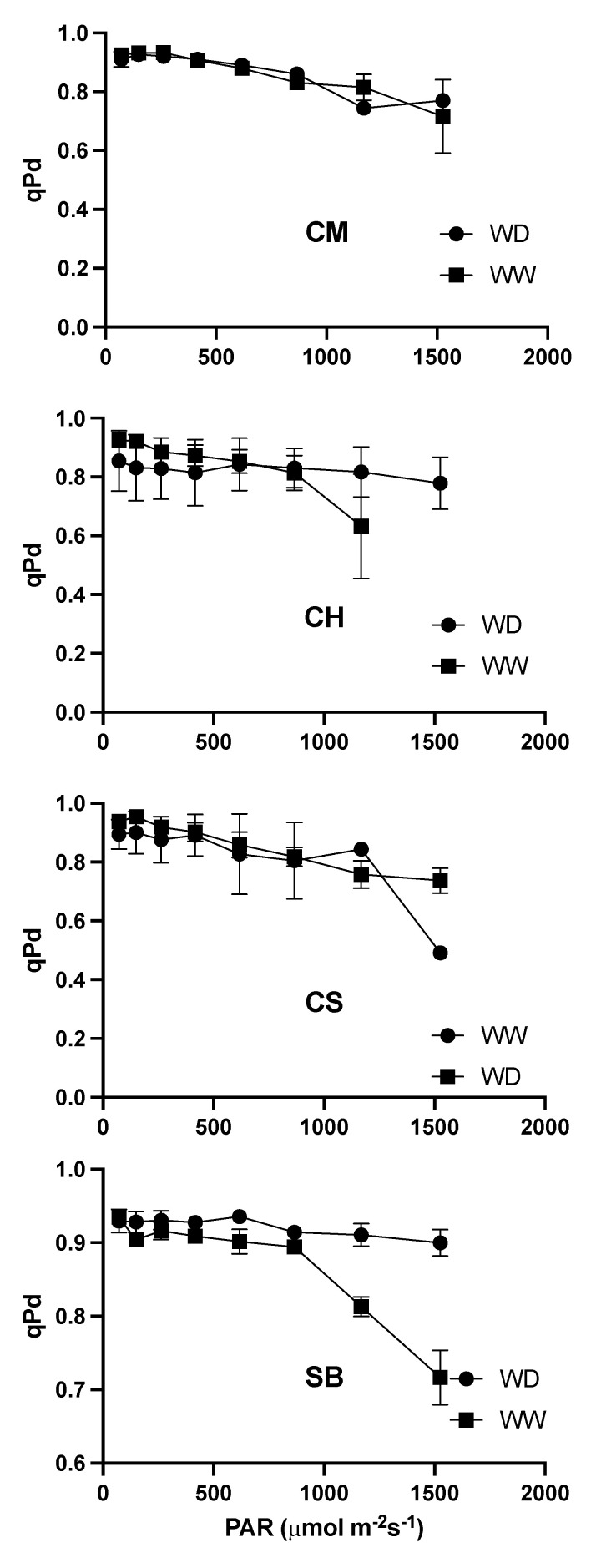
Photochemical quenching in darkness (qPd) upon increasing light intensities for WW (squares) and WD (circles) in Carmenere (CM), Chardonnay (CH), Cabernet sauvignon (CS) and Sauvignon blanc (SB). Error bars represents SE.

The qP values in the dark, immediately after an illumination period, denoted as qPd, deviated from the maximum at around 500 μmol photons m^−2^s^−1^ to 700 μmol photons m^−2^s^−1^ in all the varieties, with no differences between WW and WD plants, except in some cases at high light intensities, such as in CS and SB, but without a consistent pattern regarding the irrigation regime (Figure 9).

**Figure 10:**
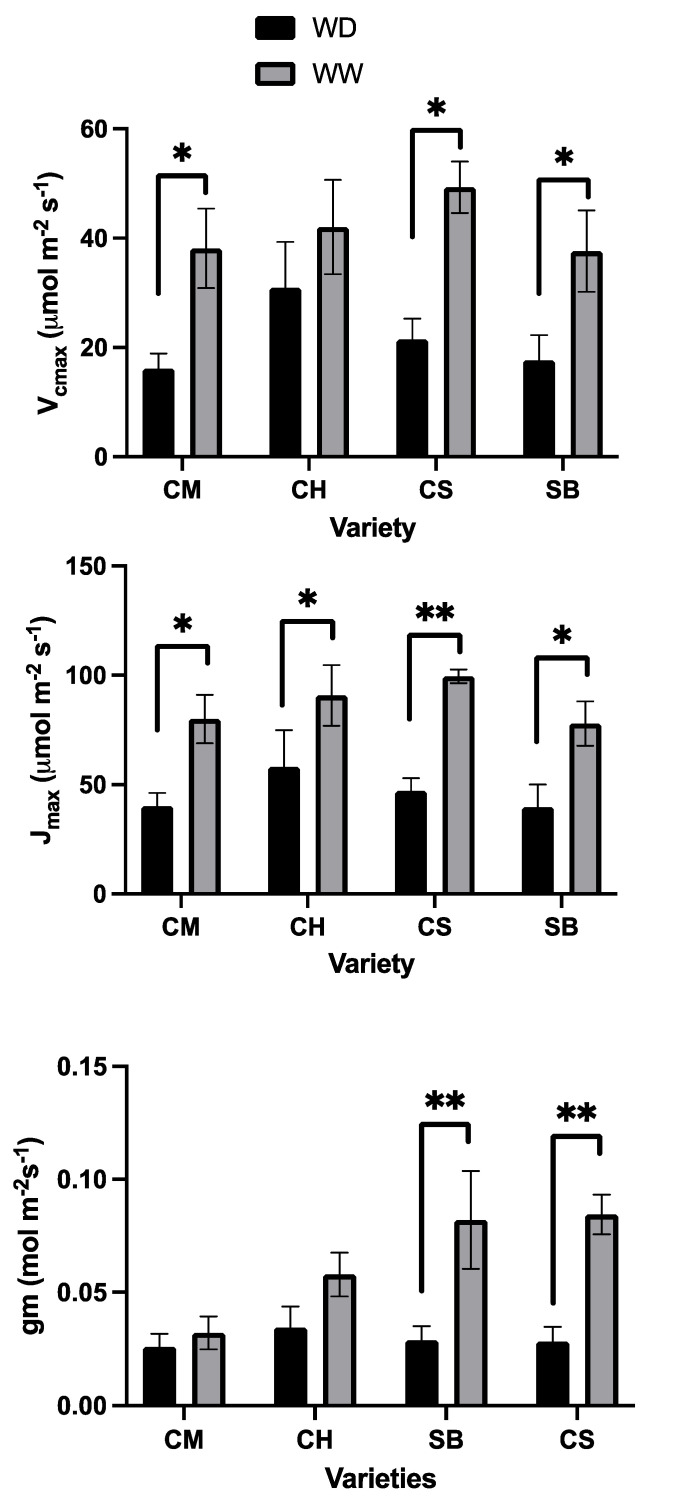
Maximum capacity for carboxylation (Vcmax, upper panel), maximum capacity for electron transport (Jmax, middle panel) and mesophyll conductance (gm, lower panel) for Carmenere (CM), Chardonnay (CH), Sauvignon blanc (SB) and Cabernet sauvignon (CS) in WD (black) and WW (grey). Error bars represent SE. Single asterisk represents significant differences between treatments for each variety *p* ≤ 0.05. Double asterisk represents significant differences between treatments for each variety *p* ≤ 0.01.

On the other hand, on average, V_cmax_ was reduced by the WD treatment, and differences were significant in CM, CS and SB, but not in CH (Figure 10). As for J_max_, values were also lower in WD plants, but in this case, differences were significant in all the varieties (Figure 10). When observing the effect of WD on gm, significant differences were observed only in SB and CS where, in both cases, the gm values in WW plants were higher on average compared to CM and CH (Figure 10).

### 2.3. Photosynthetic Pigments

**Figure 11:**
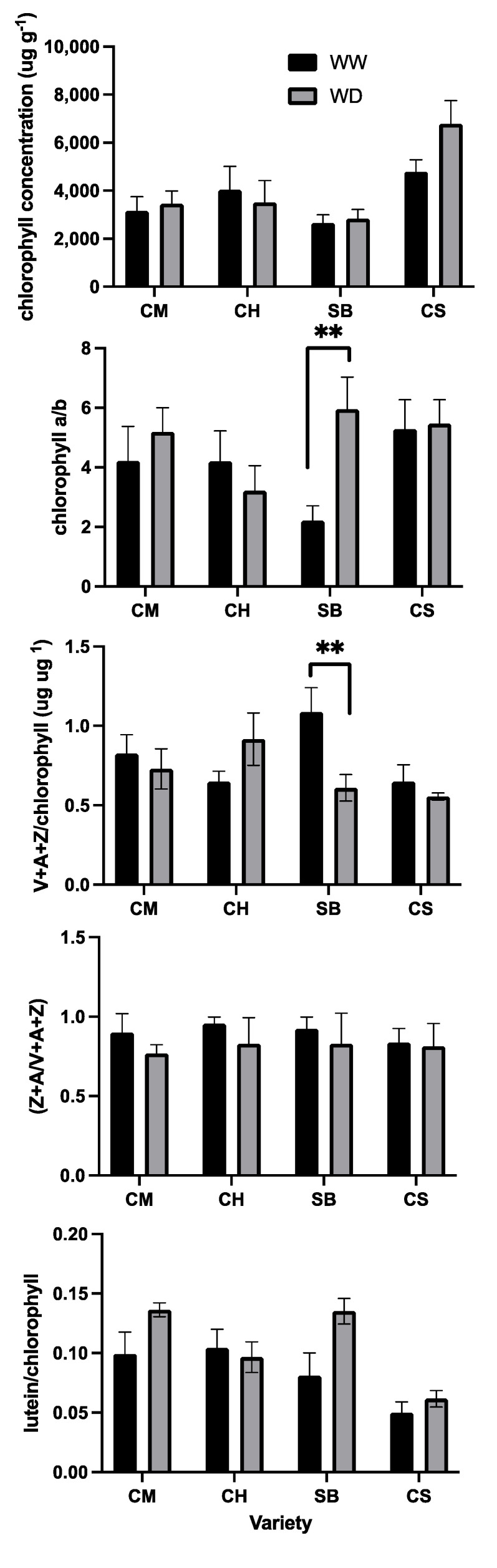
Pigment concentration in leaves, from top to bottom: Chlorophyll concentration; chlorophyll a to b ratio; the sum of violaxanthin (V), antheraxanthin (A) and zeaxanthin (Z) normalized by chlorophyll concentration, de-epoxidation state as Z+A/V+A+Z and finally lutein normalized by chlorophyll, for Carmenere (CM), Chardonnay (CH), Sauvignon blanc (SB) and Cabernet sauvignon (CS). WW in black bars and WD in grey bars. Error bars represent SE: Double asterisk represents significant differences between treatments for each variety *p* ≤ 0.01.

According to data from Figure 11, there were no differences in the total chlorophyll concentration in leaves between treatments in any of the varieties, and SB was the only variety with an increase in the chlorophyll a/b ratio in WD plants compared to those under WW conditions (Figure 11). As for the pigments involved in heat dissipation from LHCII antenna, again only in SB were differences observed between irrigation treatments, where WW resulted in a higher content compared to WD (Figure 11). Yet, the de-epoxidation index between WW and WD was similar in all the varieties (Figure 11).

### 2.4. Incident Light

**Figure 12:**
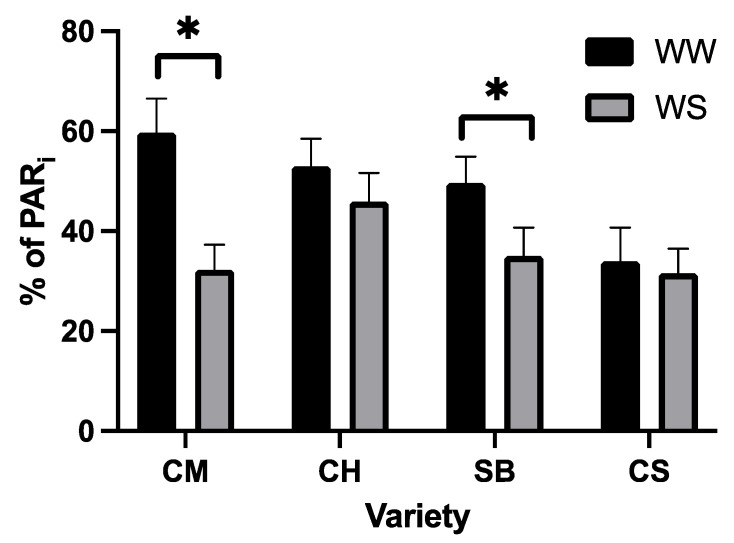
Incident light on leaves measured at midday, as a percent of the incident light above canopy (2350 mol photons m^−2^s^−1^) in Carmenere (CM), Chardonnay (CH), Sauvignon blanc (SB) and Cabernet sauvignon (CS). WW, black bars and WD, grey bars. Error bars, represent SE. Single asterisk represents significant differences between treatments for each variety *p* ≤ 0.05.

Finally, the light interception—assessed by means of positioning the PAR sensor imitating the leaf angle—is shown in Figure 12. The light intensity above the canopy at the time of the measurements was 2300 μmol photons m^−2^s^−1^. Two varieties, CM and SB, reduced the light interception at the leaf level upon WD conditions. CH also maintained the light interception upon WD at a similar value to the WW plants, in both cases at levels higher than 55% of the incident light above canopy, on average, which corresponded to 1000 μmol photons m^−2^s^−1^. As for CS, no differences in the light interception were observed between WW and WD but, in both cases, incident light was as low on average as WD plants of CM and SB (Figure 12). Intercepted light in those varieties was about 30 to 35% of the incident light above canopy, corresponding to 700 μmol photons m^−2^s^−1^ to 750 μmol photons m^−2^s^−1^ on average.

## 3. Discussion

Water loss and carbon gain are tightly bound processes, but the latter is strongly dependent on the former [31]. In general, drought would induce responses at the stomatal level, leading to photosynthetic limitations. Beyond controversies on the fact that the stomatal sensitivity to drought is not strict [22], it is well accepted that in various plant species, as well as in grapevines, differences occur in the drought thresholds upon which gs responds [20]. These lead to the question about the implications of the differential stomatal sensitivity on the mechanisms involved in excess energy dissipation and eventual photoinhibition. Here, we have assessed such responses working with four different grapevine varieties.

Each irrigation treatment implemented in the present study resulted in a roughly comparable water content within each irrigation regime, and significantly different between them (Figure 2), and even though replenishing water up to either 100% FC in the case of WW and up to 90% FC for the WD might not seem extremely different, they resulted in significant differences in the water status of the plants (Figure 3, Table 1). On one hand, the Ψ_pd_ assessed in the experimental midterm corresponded to no water deficit in WW and from weak to moderate water deficit in WD, as described before [32]. According to the same authors, the Ψ_stem_ observed during the experimental midterm as well as at the end of the experimental period also corresponds to no water deficit for WW plants and from weak to moderate deficit in the WD plants.

When observing the impact of WD in gs and AN, differences were clear between varieties (Figure 4, Table 1). First, the extent of the difference in gs and AN between WW and WD at high light intensity was higher in CS, followed by SB and CM and minimal in CH (Figure 2, Table 1). CS is known to be a highly stomatal-sensitive variety in terms of water deficit [22] and is also known to be a progeny of SB [33], which follows in the stomatal sensitivity suggested by our results. Less well-known is the CM stomatal sensitivity, even though it has been shown to be more responsive to VPD than to soil Ψ [22]. As for CH, this variety has been consistently found to behave as a low stomatal sensitive [23,34]. From our results, therefore, CS and SB are more sensitive to WD than CM and CH at the stomatal level. It must be said that Ψ_stem_ at 10 and 17 days from the irrigation treatments were different between varieties, resulting in more positive values in CH and CM (Table 1) even though the average water content along the experimental period was very similar between them (Figure 2). The range of Ψ_stem_ values are also very close and generally positive, so as to be relevant in gs differential responses. In fact, it has been shown that at such levels, gs is more responsive to VPD than the leaf and/or stem water potential [35]. The fact that plants were growing in a restrictive substrate volume might also have exacerbated the varietal sensitive responses at the stomatal level.

In general, the stomatal sensitivity to water deficit implies that more sensitive plants experience greater changes in gs than in the Ψ_stem_ and, on the contrary, less sensitive plants are prone to faster reductions in Ψ_stem_ [22], and also tend to deplete the substrate’s available water more rapidly [23]. In the present study, as already mentioned, since plants were constantly irrigated, no big changes in Ψ_stem_ were observed between treatments ranging from weak to moderate water deficit, and they were not necessarily correlated to the stomatal sensitivity.

Regarding the question about the use of light in relation to the stomatal sensitivity under mild water stress, the extent of photorespiration is strongly associated with such traits. From our data, photorespiration saturates at rather lower light intensities than A_N_, and both processes are reduced under WD. However, in the stomatal-sensitive varieties, photorespiration is reduced less than proportional to reductions in AN (Figure 6), suggesting that under stomatal limitations, photorespiration becomes an important alternative for the use of the absorbed light. Photorespiration has attracted interest mainly because of its implications in lowering A_N_, which is interpreted in many aspects as wasteful [18]. Intuitively, however, since nearly 90% of plant species on earth correspond to the C3 photosynthetic type [36], photorespiration should be thought of as a valuable feature. Indeed, more recently photorespiration has also been viewed as a relevant process because of its integration to nitrogen metabolism, sulphur assimilation and its importance in maintaining the redox balance of plants, among other features [37,38]. Moreover, reports suggest that photorespiration is highly active in environments with fluctuating light intensities, counteracting limitations to carbon fixation induced by restrictions in stomatal and mesophyll conductance [39], similar to what is observed in the more stomatal-sensitive varieties in the present study (Table 1, Figure 10).

The importance of photorespiration under water stress was demonstrated before in barley mutants [17]. Mutants with a reduced activity of photorespiratory enzymes had lower rates of photosynthesis than wild type, but also increased radiation-less energy dissipation as qN. Additionally, in grapevines, photorespiration was shown to increase in water-stressed CS plants [40], and it was associated with higher activity in the less stomatal-sensitive variety [21]. Both studies suggested a photoprotective role of photorespiration as deducted from increases in qN when suppressed. Energy dissipation associated with qN has been largely studied for decades [41] and is generally accepted as an important photoprotective mechanism in higher plants [42]. From our data, qN becomes more light sensitive upon a mild water deficit condition, and yet it is not enough to keep PSII reaction centres open to an extent similar to sufficient irrigation.

It must be underlined that the redox state of PSII, resulting from the balance between light absorption by the chlorophyll antenna and the capacity for its use on electron transport, is tightly related to the probability for photoinhibition. Besides, it is the PSII complexes that are more susceptible to photodamage, particularly in abiotic stress conditions [43], even though damage to PSI has been demonstrated under some specific environmental situations [44]. A pioneering work seeking to identify thresholds for photoinhibition based on qP proposed that any light condition sustaining reductions in open PSII reaction centres that were higher than 40% would result in long-term effects on the capacity to recover the PSII maximum quantum yield [45]. From our results, in WW conditions, a 50% reduction in qP occurs at light intensities higher than 1000 μmol photons m^−2^s^−1^ and at about 600 μmol photons m^−2^s^−1^ in WD, but if photorespiration is suppressed in the latter, only 350 μmol photons m^−2^s^−1^ to 500 μmol photons m^−2^s^−1^ is needed, a condition where qN is already saturated (Figure 7). On the other hand, not only is qP reduced more than is proportional when photorespiration is suppressed upon increases in light intensities, but also, such a response is further pronounced in the more stomatal-sensitive varieties under WD (Figure 8, Table 2). These results are a further indication that photorespiration is a relevant process in mitigating the impact of a more stomatal-sensitive response to mild water stress.

A recent approach for the identification of the light-intensity threshold upon which qN is no longer photoprotective has been proposed, and it consists of monitoring the extent of qP in the dark (qPd), enabling the detection of early signs of photoinhibition [46]. The first reports suggest that light intensities of around 1500 μmol photons m^−2^s^−1^ are necessary for an important decline in qPd, assessed in wild-type *Arabidopsis* leaves [47], which is much higher than that which we found in the present study (Figure 9). Even though we cannot be certain of the reasons for such discrepancy, in our study we roughly set the light-intensity limits in the range between 500 μmol photons m^−2^s^−1^ to 700 μmol photons m^−2^s^−1^, which coincides with that needed to saturate qN under photorespiratory conditions in WD, but exceeds such a threshold if photorespiration is suppressed (Figure 9).

Xanthophylls play important roles in photoprotection, on one hand, because of their capacity to directly quench triplet chlorophylls [47,48] and, on the other, because of their involvement in the proton motive force-dependent formation of the rapidly relaxing component of qN [49]. The so-called xanthophyll cycle results from the reversible protonation of the chloroplast lumen mediated by the activation of the violaxanthin de-epoxidase, which catalyses the de-epoxidation of violaxanthin and antheraxanthin to zeaxanthin [50], and the de-epoxidation state has been shown to be important for allosteric regulation of qN [51,52]. From our results, however, the de-epoxidation state of the xanthophyll cycle pigments was no different between irrigation regimes for any of the varieties, regardless of their stomatal sensitivity (Figure 11). Additionally, the total pool of xanthophylls cycle pigments normalised by the total chlorophyll concentration did not change upon mild water deficit, except for SB, but increased upon WD (Figure 11). This is similar to previous reports upon mild water stress in a different plant species where the xanthophyll cycle pool was not altered [53], even though there are reports that associate an increase in the total xanthophylls under conditions of excess absorbed energy as concomitant to a higher capacity for qN [50,54]. As for lutein, which has been proposed as a xanthophyll pigment involved in qN and acting as a direct quencher from chlorophyll a (Ruban et al., 2007), no changes upon mild water stress were observed, expressed on a chlorophyll basis (Figure 11).

An additional photoprotective strategy in plants consists of the modification of the chlorophyll pigment concentration [55,56], which could be associated with changes in both the PSII/PSI reaction centres ratio and PSII antenna size. These may be assessed indistinctively through the chlorophyll a/b ratio. In general, under environmental conditions exacerbating the light-energy pressure on PSII reaction centres, reductions in the total chlorophyll concentration have been observed [55], as well as increases in the chlorophyll a/b ratio [57], particularly in grapevine leaves as they grow in the season experiencing constraining conditions [58]. These strategies were clearly not important as a response to mild water stress in any of the grapevine varieties, except for SB (Figure 11). Therefore, adaptation to a mild water-stress condition does not seem to be associated with a consistent response at the photosynthetic pigment level, at least in terms of xanthophylls and chlorophylls.

Finally, we tested the hypothesis that the grapevines respond to mild water stress, avoiding light, as a strategy for decreasing the excitation pressure on PSII reaction centres induced by water stress. Indeed, light avoidance has been reported to be a strategy to escape from light and heat [59] mainly described in leguminous species as paraheliotropism, a short-term reversible light avoidance leaf movement [28,29]. More recently, however, it has been documented that leaf angle changes in a non-reversible manner in the grapevine Aleatico when plants experience water stress, and also that such light avoidance correlates with the extent of the reduction in stomatal conductance [30]. We measured the incident light on the leaf lamina and found that in CM and SB, it was reduced under WD, but not in CH nor in CS (Figure 12). In the latter, however, even well-watered plants had low incident light. From here, the least stomatal-sensitive variety maintained the light interception when in mild water stress, whereas the more sensitive one had a low average light interception under both water conditions. The intermediate varieties, on the other hand, reduced the light interception under moderate water stress. Interestingly, the light interception at midday in those varieties avoiding light under water stress reached about 700 μmol photons m^−2^s^−1^ to 750 μmol photons m^−2^s^−1^, which corresponds to 35% of the incident light above the canopy (Figure 12). This is very close to the threshold from which qN is no longer photoprotective (Figure 9), and upon which 50% of qP reduction is observed in photorespiratory air (Figure 7). Our data support the evidence of a relationship between leaf-light interception and stomatal conductance [30] and further suggest that the extent of the avoidance matches the maximum light-intensity needed for safe photosynthetic activity.

## 4. Materials and Methods

### 4.1. Plant Material

One-year-old Cabernet Sauvignon (CS), Carménère (CM), Chardonnay (CH) and Sauvignon Blanc (SB) plants grafted on 110R rootstock were planted in 20 L pots in a mixture of pit, coconut peel and perlite (40/20/20 *w*/*w*) from Deitan (Deitan solutions Co), during winter 2019. Three to four buds sprouted from each plant. The dry weight and weight at field capacity of each pot was recorded. Every pot was irrigated up to field capacity (FC), three times a week, by weight, until midsummer, and fertilised with complete Hoagland solution once a week. Plants were allowed to grow without trellising, with main and secondary shoots. Then, 5 plants from each variety continued with full irrigation and 5 were irrigated only up to 90% FC, three times a week, for three weeks.

### 4.2. Leaf Water Potential

Leaf water potentials (Ψ) were assessed in leaves positioned at the middle of each main shoot, well exposed to sunlight. Predawn leaf water potential (Ψ_pd_) and midday stem water potential (Ψ_stem_) were measured by means of a pressure chamber (PMS Instrument Company, Model 615, Albany, OR, USA). Ψ_pd_ was only measured 10 days from the beginning of the irrigation treatments and the Ψ_stem_ was measured on that same date and, additionally, at the end of the experiment, 21 days after the beginning of the irrigation treatments. For Ψ_stem_ and Ψ_pd_, one leaf per plant was used. The procedure was as described in [22]. Briefly, the leaves were placed into the pressure chamber with the petiole protruding from the chamber lid. The chamber was pressurised using a nitrogen tank, and Ψ was recorded when the initial xylem sap was observed emerging from the cut end of the petiole using a stereo microscope (model V424B, Omax, https://omaxmicroscope.com/, accessed on 29 February 2022). The predawn water potential (Ψ_pd_) was measured before sunrise between 5:00 h and 7:00 h. The leaves were wrapped in a damp paper towel, bagged, detached with a fresh razor blade, transported in a fresh cooler box and leaves were pressurised until two minutes after detachment. The Ψ_stem_ was assessed between 11:15 h and 12:45 h. For Ψ_stem_, leaves were previously enclosed in aluminised plastic bags at least 2 h before measurement, and leaves were detached from their shoot immediately after gas-exchange measurement, transported in a fresh cooler box, and finally pressurised 3 min after detachment.

### 4.3. Maximum Capacity for Carboxylation (V_max_), Maximum Electron Transport Capacity (J_max_) and Mesophyll Conductance (gm)

The parameters Vmax, Jmax and gm were calculated from the response of photosynthetic assimilation to varying intercellular partial pressure of CO_2_, according to [60] and by means of rapid ACi response (RACiR) curves, using the LI-6800 Portable Photosynthesis System equipped with the Multiphase Flash Fluorometer and Chamber (LI- COR Inc., Lincoln, NE, USA), with corrections and protocols as in [61]. Leaves attached to plants were placed inside the chamber at 420 ppm CO_2_ and left to acclimate for 5 min. The auto control function of the LI-6800 was used to program a “down” ramp from 420 to 20 ppm at a rate of 200 ppm min^−1^ of CO_2_, immediately followed, 10 to 15 s later, by an “up” ramp from 20 to 1520 ppm at a rate of 100 ppm min^−1^. Recordings were set every 2 s. The reference and sample infrared gas analysers (IRGAs) were matched before the start of each curve and only the data collected from the “up” ramps (20 to 1520 ppm) were used to establish the CO_2_ response curves. The raw data from these “up ramps” were filtered automatically using a delta threshold value (±0.05, A_Ni_ − A_Ni−1_) to keep only the quasi-linear portion of the data, where the chamber mixing was at steady state, also removing outliers. The raw data obtained from the RACiR curve were corrected to account for measurement lags between the reference and sample [CO_2_], match offsets and system residual time delays. For these, data collected from the quasi-linear portion of the RACiR curve measured with the chamber empty (ECRC) were used following [61]. Each set of response-curve data was corrected using empty chamber data obtained the same day. The maximum rate of carboxylation (V_cmax_) and the maximum rate of electron at PAR = 1261 µmol m^−2^s^−1^ (J_max_) were estimated from the A-Ci curve data using the R ‘plantecophys’ package, adjusting a bilinear fitting method [62].

The mesophyll conductance (gm) was estimated using the “Variable J” method [63] by combining gas exchange and chlorophyll fluorescence, according to the equation:

(1) $$\mathrm{gm}=\frac{A_{N}}{C_{i}-\frac{\Gamma^{*}\left[\mathrm{ETR}+8\left(A_{N}+R_{\mathrm{day}}\right)\right]}{\mathrm{ETR}-4\left(A_{N}+R_{\mathrm{day}}\right)}}$$

where gm is the mesophyll conductance, A_N_ is the net CO_2_ assimilation, Ci is the intercellular CO_2_ concentration, R_day_ is the mitochondrial respiration in light and Γ* is the CO_2_ compensation point in the absence of R_day_. ETR is the electron transport rate. Γ* and R_day_ were estimated according to Walker et al. (2016), using three slopes of A_N_-Ci under low light and low CO_2_ concentrations. In theory, three CO_2_ response curves obtained by varying CO_2_ concentrations from 150 to 50 μmol CO_2_ mol^−1^ under three PPFDs (for CH and CM were 421, 210 and 42 μmol fotons m^−2^s^−1^ and for CS and SB 421, 210 and 63 μmol fotons m^−2^s^−1^) would intersect with each other at a point, and the intersection point at *x*-axis and *y*-axis were considered to Γ* and R_day_, respectively. However, in practice, these three linear regressions of the intersected A_N_-Ci curves formed a triangle range rather than a single point, and the Γ* and R_day_ were calculated as the barycentre of the triangle formed by the intersection of the three lines at the *x*-axis and *y*-axis.

### 4.4. Gas Exchange and Chlorophyll Fluorescence, qPd

The A_N_ vs. PAR response curves were performed with an LI-6800 Portable Photosynthesis System equipped with the Multiphase Flash Fluorometer. Plants were taken into the lab, and a leaf from the middle section of a main stem was dark adapted by covering the lamina with aluminium foil for 40 min. The leaves were then placed into the leaf chamber, avoiding illumination and dark respiration, and chlorophyll fluorescence (Fm and Fo) was recorded immediately after reaching a steady state of gas exchange and chl fluorescence. Then, leaves were acclimated in the chamber for 30–40 min at 1500 μmol photons m^−2^s^−1^, waiting to achieve a steady state for gas exchange and chl fluorescence again. Thereafter, gas exchange followed by chlorophyll fluorescence measurements was recorded at 3–5 min intervals at the following decreasing irradiance steps: 1500, 1200, 900, 600, 300, 150 and 50 μmol photons m^−2^s^−1^. Later, for the measurements under non photorespiratory conditions, we followed previously described protocols, as in [64,65]. Briefly, a gas N_2_ mixture with O_2_ lower than 1% was inflowed through the inlet of the LI-6800-F using a three-way valve until saturating the chamber with the inert gas. After reaching a steady state of gas exchange, representing gross net CO_2_ assimilation (A_G_), gas exchange followed by chlorophyll fluorescence measurements was recorded at 3–5 min intervals at the following increasing irradiance steps: 50, 150, 300, 600, 900, 1250 and 1500 μmol photons m^−2^s^−1^ to obtain the light response curve under non-photorespiratory conditions. Photorespiration (P_hresp_) resulted from the deduction of A_G_ and A_N_. For all measurements, the chamber conditions for the light response curves were the following: 400 mL L^−1^ of CO_2_, flow 600 mmol s^−1^, leaf-vapour pressure difference 1.8 kPa and temperature 28 °C.

The light response curve was fitted using non-rectangular hyperbola model with four parameters:

$$\mathrm{An}=\frac{\Phi *\mathrm{PPFD}+\mathrm{Amax}\;-\sqrt{{\left(\Theta \;\mathrm{PPFD}+\mathrm{Amax}\right)}^{2}-4\;\Theta \;\Phi \;\mathrm{PPFD}\;\mathrm{Amax}}}{2\Theta }-\mathrm{Rd}$$

were, Amax (max gross photosynthetic rate), Rd (dark respiration), F (apparent quantum yield), Q (curvature parameter, dimensionless) from Marshall and Biscoe (1980).

The chlorophyll fluorescence parameters, qP and qN, were also obtained as follows:

$$\mathrm{qP}=\frac{{\mathrm{fm}}^{\prime }-\mathrm{fs}}{{\mathrm{fm}}^{\prime }-{\mathrm{fo}}^{\prime }}\;$$

and

$$\mathrm{qN}=\frac{\mathrm{fm}-{\mathrm{fm}}^{\prime }}{\mathrm{fm}-{\mathrm{fo}}^{\prime }}$$

where fm was recorded with dark respiration measurement and fs, fm′ and fs′ were obtained at each step of irradiance.

The assessment of qP in darkness was carried out as described in [46], by means of a modulated fluorimeter (Hansatech, FMS2, Norfolk, UK). Plants were taken into the lab, and one leaf per plant was dark adapted for 40 min with an aluminium bag. A monitoring leaf-clip was placed in the dark-adapted leaf, and the F_0_ measurement was recorded in the presence of low intensity far red light followed by a high intensity saturating light pulse for F_m_. A script was programmed with increasing light intensities from 60, 100, 250, 400, 600, 850, 1150 and 1500 μmol photons m^−2^s^−1^. Each illumination period lasted 5 min with saturating pulses at the second and fifth minutes for NPQ calculation and immediately after the second pulse, the light was switched off. After 7 s of far-red illumination, a saturating pulse was applied for 5 s, followed by the next cycle of actinic illumination. Fo´_calc._ and qPd were calculated as in [46].

### 4.5. Pigments

At the end of the experiment, two leaves per plant were detached from the middle of the main stem, and immediately frozen in liquid nitrogen and later stored at −80 °C. The protocol for the determination of xanthophylls and chlorophylls *a* and *b* was according to [66], but with minor modifications. All solvents used during sample extraction, preparation and analysis were HPLC grade, purchased from Merck. The standards trans-*b*-carotene, chlorophyll a and b, the xanthophylls zeaxanthin, antheraxanthin, and violaxanthin, and the internal standard (IS), ß -apo-caroten-8-al, were purchased from Sigma-Aldrich. Leaf samples were grinded in liquid nitrogen in a mortar and pestle and 300 mg was suspended in 50 mL acetone, then concentrated using a rotary evaporator at 37 °C for 10 min. The sample was resuspended again in 2 mL of acetone, and an internal standard consisting of ß-apo-caroten-8-al was added, transferring the sample to a 2 mL Eppendorf. Subsequently, the tubes were vortexed for 30 min, followed by separation of the tissue debris by centrifugation (11000 g, 3 min). A 300 µL aliquot of the acetone extract (now containing pigment) was extracted and added to 1 mL of extraction buffer and vortex washed for 5 min. Ethyl acetate (200 µL) was added, vortexed briefly, and centrifuged (11000 g, 5 min) to split the mixture. A 50 µL aliquot of the upper ethyl acetate phase (containing the pigment) was extracted, added to 200 µL of methanol containing 0.125% (*w*/*v*) BHT, and 200 µL was transferred to amber HPLC vials (containing 200 µL vial inserts) and sealed.

For the chromatographic analysis of xanthophylls and chlorophylls, all pigments were separated by RT-HPLC on an Agilent 1260 Infinity HPLC system, equipped with a DAD detector. A LiChrosorb^®^ RP-18 HPLC Column (250 mm × 4.6 mm, particle size 5 μm) and guard cartridge from Merck KGaA, Germany (Darmstadt, Germany) were used. In order to separate the major pigments extracted from the grapevine tissue, a ternary mobile phase of methanol (solvent A), methanol containing 80/20 (*v*/*v*) ammonium acetate 0.5 N (solvent B) and tetrahydrofuran (solvent C) was employed. The flow rate was 1 mL min^−1^ at 25 °C. The elution program was isocratic at 100% B for 5 min, followed by an increase to 98% A and 2% C for 12.2 min. C increased to 20% and A decreased to 80% for 8.8 min, followed by an increase of A to 98% and a decrease of C to 2% for 8 min, followed by isocratic of B to 100%. The column was equilibrated for 1 h at the initial conditions before each injection. The injection volume was 20 µL.

### 4.6. Incident Light on Leaves

In order to assess the incident light on leaves, as a proxy for light interception, a quantum radiometric probe sensor was used (Delta Ohm, Padua, It). Measurements consisted of positioning the PAR sensor imitating the leaf angle of leaves from 10 consecutive nodes, in the middle part of each main stem per replicate. The measurements were performed at the end of the experiment, at 13:00 h, when incident light measured parallel to the ground was at the maximum, equivalent to 2350 μmol photons m^−2^s^−1^.

### 4.7. Statistical Analysis and Experimental Design

The experiment consisted of two treatments of irrigation, for four varieties, with five replicates each. Each variety growing in a 20 L pot was arranged on a row, alternating WW and WD. Rows were 1.5 m apart, without shading from other plants, and each pot was distanced 1.5 m on the row. The paired comparisons and SE intervals, as well as plots, were obtained using Prism 9, except for A_N_ responses to light and CO_2_, which were obtained using the Plantecophys-An R package [67].

## 5. Conclusions

In order to assess stomatal sensitive/non-sensitive grapevine varieties’ photosynthetic responses to drought, we assessed the photorespiratory activity, the non-photochemical energy dissipation and the associated concentration of open reaction centres, as well as the responses at the level of photosynthetic pigments and the incident light at the leaf level in CM, CH, CS and SB. We worked with one-year-old potted plants that were either well-watered or had a mild water deficit. From our results, we conclude that photorespiration is an important biochemical pathway in grapevines, but to a greater extent in the varieties with higher stomatal sensitivity, accounting for a 75% of A_N_ under mild water stress. This is at least double that in the less stomatal-sensitive variety. Additionally, the importance of photorespiration is clear under mild stress since it alleviates the energy pressure on PSII, again to a greater extent in the more stomatal-sensitive varieties. Importantly, photorespiration allows qN to saturate at light intensities slightly lower than the limit from which PSII reaction centres are under the risk of photoinhibition. Such a light-intensity limit is further secured under mild water stress by reducing the incident light, likely with a modification of the leaf angle, except for the less stomatal-sensitive variety, which seems to take risks. Even though the non-photochemical energy dissipation plays its part, increasing under mild water stress at high light, the concentration of the pigments involved such as xanthophylls and lutein remains constant. Additionally, there is no indication of a consistent acclimation at the level of chlorophyll pigments. We must underline, though, that our results are not necessarily a prediction of the varietal responses of grapevines to mild water stress in field conditions, since root volume, root to shoot ratio as well as specific rootstock to scion interactions are expected to occur differently than those in one-year-old potted plants.

## Figures and Tables

**Table 1:** Gas Exchange and water potential values of grapevine cultivars under two irrigation treatments. An, assimilation rate, gsw, stomatal conductance, Y_pd 10_, pre-dawn water potential at 10 days after irrigation treatments started and Y_md_, stem water potential at midday at 10 and 17 days from the water treatments were imposed.

	Y_pd 10_		Y_md 10_		Y_md 17_		gsw		A_n_	
	(MPa)		(MPa)		(MPa)		(mmol H_2_O m^−2^s^−1^)		(mmol CO_2_ m^−2^s^−1^)	
***Cultivar***										
CH	−0.21 ± 0.1		−0.75 ± 0.21 a		−0.67 ± 0.20 a		107 ± 53 a		7.96 ± 4.04	
CM	−0.25 ± 0.13		−0.73 ± 0.18 a		−0.78 ± 0.29 ab		59 ± 27 b		6.25 ± 3.12	
SB	−0.25 ± 0.13		−0.97 ± 0.19 b		−0.93 ± 0.32 b		72 ± 33 ab		6.36 ± 3.96	
CS	−0.23 ± 0.14		−0.93 ± 0.28 b		−0.92 ± 0.36 b		83 ± 41 a		8.30 ± 4.23	
***Trat***										
WW	−0.12 ± 0.03		−0.69 ± 0.12		−0.58 ± 0.12		106 ± 32		9.76 ± 2.87	
WD	−0.34 ± 0.07		−1.02 ± 0.2		−1.09 ± 0.22		56 ± 37		4.68 ± 2.87	
***Cultivar × Trat***	WW	WD	WW	WD	WW	WD	WW	WD	WW	WD
CH	−0.11	−0.3	−0.65	−0.87	−0.51	−0.86	128	87	9.81	6.12
CM	−0.13	−0.34	−0.58	−0.88	−0.53	−1.02	77	36	8.05	4.45
SB	−0.13	−0.36	−0.81	−1.12	−0.68	−1.18	100	50	9.14	3.58
CS	−0.10	−0.36	−0.71	−1.21	−0.6	−1.24	118	47	12.02	4.57
***Fixed Effects***										
*Cultivar*	0.2419		0.0002		0.001		0.0114		0.2563	
*Trat*	<0.0001		<0.0001		<0.0001		<0.0001		<0.0001	
*Cultivar × Trat*	0.4667		0.1275		0.2061		0.2933		0.3874	

Values correspond to mean ± SD. Also, for *p*-values < 0.05 for fixed effects, post hoc Tukey’s honestly were tested. Different letters indicate significative differences between means.

**Table 2:** Relationship between qP under non-photorespiratory conditions and photorespiratory conditions of CM, CH, CS and SB under WD and WW.

qP (no Phresp)/qP.		
***Cultivar***		
CM	0.59	
CH	0.75	
CS	0.47	
SB	0.52	
***Trat***		
WW	0.46	
WD	0.70	
***Variety × Trat***	WW	WD
CM	0.59	0.63
CH	0.75	0.83
CS	0.47 a	0.60 b
SB	0.52 a	0.75 b
***Fixed effects***		
*Variety*	0.016	
*Trat*	0.0004	
*Variety × Trat*	0.22	

Values correspond to mean. Also, for *p*-values < 0.05 for fixed effects, post hoc Tukey’s honestly were tested. Different letters indicate significative differences between means.

## Data Availability

Data are available from the authors upon request.
