# Correction: Villalobos-González et al. Photoprotection Is Achieved by Photorespiration and Modification of the Leaf Incident Light, and Their Extent Is Modulated by the Stomatal Sensitivity to Water Deficit in Grapevines. *Plants* 2022, *11*, 1050

**DOI:** 10.3390/plants11162096

**Published:** 2022-08-12

**Authors:** Luis Villalobos-González, Nicolás Alarcón, Roberto Bastías, Cristobal Pérez, René Sanz, Álvaro Peña-Neira, Claudio Pastenes

**Affiliations:** Facultad de Ciencias Agronómicas, Universidad de Chile, Santiago 8820808, Chile

In the original publication [1], there was a mistake in the legend for Figure 7. The figure legend was accidentally deleted and replaced with the main text. The correct legend appears below with the correct main text in Section 2.2. Plant Water Status, Photosynthesis, Photorespiration and Chlorophyll Fluorescence: 

**Figure 7.** Photochemical (qP) and non-photochemical (qN) quenching responses of WW plants (two panels to the left) and WD plants (two panels to the right) to light intensity in photorespiratory conditions (+Phr: squares, black colour) and non-photorespiratory conditions (-Phr: circles, red colour) in Carmenere (CM), Chardonnay (CH), Cabernet sauvignon (CS) and Sauvignon blanc (SB). Error bars represent SE.

The ratio between qP under non-photorespiratory conditions vs. photorespiratory conditions is shown in Figure 8. In the case of CM and CH, no significant differences were observed between irrigation treatments. In CS, differences in light intensities were significant: 750 μmol photons m^−2^s^−1^ and higher. In SB WW and WD, the ratio was significantly different at light intensities of 250 μmol photons m^−2^s^−1^ and higher, in both cases with higher values in WD plants (Figure 8). Differences between varieties were significant, as well as between irrigation treatments, but with no interaction between both factors (Table 2).

The authors apologize for any inconvenience caused and state that the scientific conclusions are unaffected. This correction was approved by the Academic Editor. The original publication has also been updated.
